# Maternal immune activation affects socio-communicative behavior in adult rats

**DOI:** 10.1038/s41598-023-28919-z

**Published:** 2023-02-02

**Authors:** Kinga Gzieło, Diana Piotrowska, Ewa Litwa, Piotr Popik, Agnieszka Nikiforuk

**Affiliations:** Department of Behavioral Neuroscience and Drug Development, Maj Institute of Pharmacology Polish Academy of Sciences, 12 Smętna Street, 31-343 Kraków, Poland

**Keywords:** Social behaviour, Psychiatric disorders, Autism spectrum disorders, Neuroscience, Diseases of the nervous system, Autism spectrum disorders, Developmental disorders, Risk factors, Preclinical research, Experimental models of disease

## Abstract

A wide body of evidence suggests a relationship between maternal immune activation (MIA) and neurodevelopmental disorders such as autism spectrum disorder (ASD). Since social and communicative deficits are included in the first diagnostic criterion of ASD, we aimed to characterize socio-communicative behaviors in the MIA model based on prenatal exposure to poly(I:C). Our previous studies demonstrated impaired socio-communicative functioning in poly(I:C)-exposed adolescent rats. Therefore, the current study sought to clarify whether these changes would persist beyond adolescence. For this purpose, we analyzed behavior during the social interaction test and recorded ultrasonic vocalizations (USVs) accompanying interactions between adult poly(I:C) rats. The results demonstrated that the altered pattern of social behavior in poly(I:C) males was accompanied by the changes in acoustic parameters of emitted USVs. Poly(I:C) males also demonstrated an impaired olfactory preference for social stimuli. While poly(I:C) females did not differ from controls in socio-positive behaviors, they displayed aggression during the social encounter and were more reactive to somatosensory stimulation. Furthermore, the locomotor pattern of poly(I:C) animals were characterized by repetitive behaviors. Finally, poly(I:C) reduced parvalbumin and GAD67 expression in the cerebellum. The results showed that prenatal poly(I:C) exposure altered the pattern of socio-communicative behaviors of adult rats in a sex-specific manner.

## Introduction

Autism spectrum disorder (ASD) is a group of neurodevelopmental disorders characterized by two core symptoms: impairments in social interaction and communication and restricted, repetitive patterns of behaviors or interests^1^. ASD is a lifelong disease as the first symptoms may be detected in early childhood and typically persist into adulthood. The severity of symptoms may vary between patients. While individuals with high-functioning autism can live independent lives, some struggle with severe disabilities and require care and support. Regardless of symptom intensity, ASD significantly limits everyday functioning, particularly in the social/communication domain, where no specific and effective pharmacotherapy is currently available^2^. Therefore, to improve understanding of ASD etiopathology of this disease and to identify possible treatment options, preclinical ASD models have been developed^3^.

Although recent studies indicate a genetic basis for ASD susceptibility, environmental factors can also contribute to this disorder. Increasing evidence points to the immune system as a convergent target for gene and environment interactions that confer ASD-related risk factors^4^. While excessive or dysregulated inflammation has been implicated in the pathogenesis of several chronic diseases, gestation is the period the most susceptible to this insult. Consequently, maternal infections during pregnancy, including viral or bacterial infections, can adversely impact fetal neurodevelopment. Therefore, maternal immune activation (MIA) has repeatedly been associated with the onset of neuropsychiatric disorders such as autism or schizophrenia in humans^5^. The neurodevelopmental consequences of MIA have also been studied in animal models. One of the most used models of MIA-evoked ASD is the exposure of pregnant dams to polyinosinic:polycytidylic acid (poly(I:C)), a commercially available synthetic analog of double-stranded RNA. Poly(I:C) mimics a viral infection, leading to the disturbance of brain development and, consequently, to ASD-like behavioral abnormalities in rodents^6,7^.

Deficits in the socio-communicative domain, a hallmark of autistic symptomatology, may be successfully investigated in laboratory conditions^8^. Rodents, especially rats, are very social animals with a highly developed pattern of social behaviors directly related to conspecifics^9^. The early forms of social interactions appear already in adolescents. This typical for young rats social play behavior emerges three weeks after birth and peaks between the 28th and 40th postnatal days^10^. When the animals become sexually mature, specific playful events such as pinning and pouncing disappear. After that time, the socio-positive behavior of adult rats is characterized mainly by sniffing, anogenital sniffing, grooming, climbing, and following.

During social encounters, rats also communicate using two main types of ultrasonic vocalizations (USVs): low frequency (22-kHz) and high frequency (50-kHz) calls^11^. While 22-kHz alarm calls are associated with unpleasant events (e.g., facing the predator), 50-kHz “happy” calls are linked to rewarding events and reflect socio-positive aspects of social interactions^12^. Rats emit 50-kHz USVs also in response to playful somatosensory stimulation by an experimenter known as “tickling”^13^. A vast repertoire of rats’ 50-kHz USVs differs in their durations and patterns of frequency modulations. The most characteristic type of high frequency-modulated calls are “trills” that appear in spectrograms as rhythmic waves of ups and downs. Thus, a complex behavioral repertoire of rats accompanied by a rich acoustic communication system can serve as an excellent determinant of autism-related socio-communicative deficits^14^.

Our previous study demonstrated that prenatal poly(I:C) exposure impaired socio-communicative functioning in adolescent rats, as revealed by social play deficits together with reduced USV emission^15^. However, the question remained whether these changes would persist beyond the adolescent period. Thus, building upon our previous work in adolescent rats, the current study aimed to examine the effects of poly(I:C)-induced MIA on adult rats’ social behavior and ultrasonic vocalizations. For this purpose, we analyzed behavior during the social interaction test and recorded USVs accompanying interactions between rats. Furthermore, as tickle-evoked 50-kHz calls may be a useful behavioral marker of positive social affect in rats^16^, we also recorded USVs emitted by rats when manually tickled by an experimenter. In addition, we performed the detailed characteristics of the acoustic calls’ features that provide a more comprehensive assessment of ultrasounds emitted by rats than using only the quantitative measures. Furthermore, as there are indications that the olfactory system mediates social behavior, we also examined rats’ preference for social stimuli in the olfactory preference test^17^. Finally, to study another core symptom of ASD, i.e., repetitive behaviors^18^, the number of repetitive movements was measured using activity meters.

Evidence indicates that the prevalence of ASD in males appears to be higher than in females. The reasons for this sex ratio bias are still unknown; however, one may notice that females are at an elevated risk of missed or late diagnosis due to the sex-specific differences in the manifestation of autistic phenotypes and the females’ innate ability to camouflage ASD symptoms^19,20^. However, preclinical studies often omit females, and thus the sex differences^21^. Therefore, in this study, we focused on examining both sexes.

Although precise mechanisms leading to autistic-related abnormalities are still largely unknown, one of the most recognized theories of autism postulates that the key pathological feature of ASD is the imbalance between excitatory/inhibitory neurotransmission in the brain^22^. In line with this theory, abnormalities in inhibitory GABAergic signaling may play a role in the etiology of neurodevelopmental disorders, including autism (^23^, see details in the “Discussion” section). Hence, considering GABA system dysfunction in autism, we also studied the protein levels of parvalbumin and GAD67 in brain structures that regulate social behavior, cognition, or motor skills in rats.

## Results

### Social interaction test

**Figure 1:**
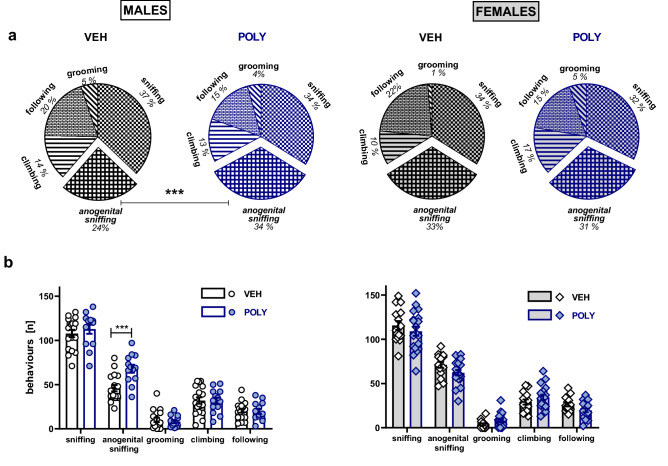
Behavior during the social interaction test. Poly(I:C) exposure increased the time (**a**) and the number of episodes (**b**) of anogenital sniffing in male rats. Data are presented as the percentage of time spent on a given behavior (**a**) and a mean ± SEM of the number of episodes of a given behavior (**b**). Symbols: ***p < 0.001, a significant difference between vehicle- and poly(I:C)-exposed males (planned comparisons).

The pattern of social behaviors differed between poly(I:C)-exposed rats and their controls in a sex-dependent manner (treatment × sex × behavior interaction: F[4,305] = 2.83, p = 0.0248; Fig. 1a, and F[4,305]  = 4.99, p = 0.0006; Fig. 1b). Planned comparisons revealed that poly(I:C)-exposed males, but not females, spent more time on anogenital sniffing (t = 3.37, p = 0.0008; Fig. 1a) and demonstrated a higher number of episodes of anogenital sniffing (t = 4.43, p < 0.0001; Fig. 1b).

**Figure 2:**
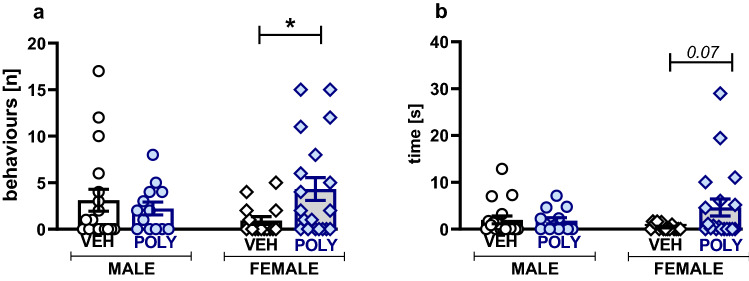
Aggressive behavior in the social interaction test. Poly(I:C) exposure increased the number and duration of aggressive episodes only in female rats. Data are presented as a mean ± SEM of the number of episodes (**a**) and time (**b**) of aggressive behavior. Symbols: *p < 0.05, a significant difference between vehicle- and poly(I:C)-exposed females (Mann–Whitney U test).

We did not observe changes in aggressive behaviors in poly(I:C) males compared to their controls (Fig. 2). However, interestingly, poly(I:C) exposure enhanced aggression in females, as revealed by the increased number of episodes of aggression (U = 87, p = 0.0394, Mann–Whitney U test; Fig. 2a). Moreover, these episodes tended to be longer than those in control females (U = 94, p = 0.0750, Mann–Whitney U Test; Fig. 2b).

### Social interaction-induced USVs

**Figure 3:**
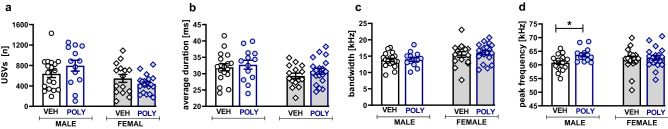
Vocalizations during the social interaction test: total USV emission. Poly(I:C) exposure did not affect the total number (**a**), average duration **(b),** and bandwidth **(c)** but increased the peak frequency (**d**) of emitted calls in males. Data are presented as a mean ± SEM. Symbols: *p < 0.05, a significant difference between vehicle- and poly(I:C)-exposed males (planned comparisons).

As illustrated in Fig. 3a, poly(I:C) exposure did not significantly affect the total number of emitted 50-kHz calls as well as their average duration (Fig. 3b) and bandwidth (Fig. 3c). However, there was a trend for the poly(I:C) group to vocalize at a higher peak frequency (treatment effect: F[1,61]  = 3.95, p = 0.0513; Fig. 3d). Accordingly, planned comparisons revealed significant increases in males (t = 2.22, p = 0.0305; Fig. 3d).

**Figure 4:**
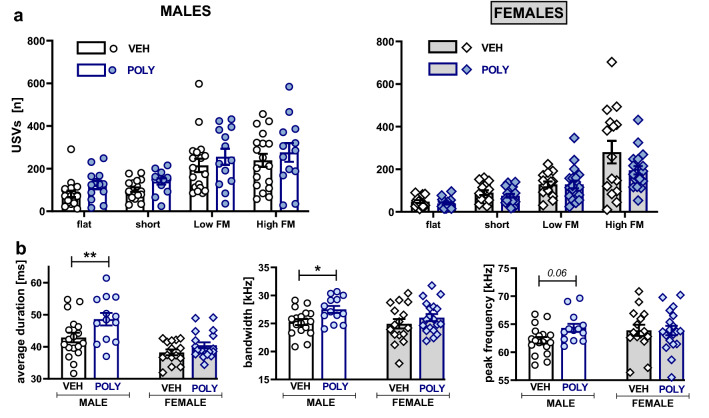
Vocalizations during the social interaction test: USV categories. Poly(I:C) exposure did not affect the number of USVs within categories (**a**) but changed the acoustic characteristics of high frequency modulated (HFM) sounds (**b**). Data are presented as a mean ± SEM of the number of a given call type (**a**) and average duration, bandwidth, and peak frequency (**b**) of the HFM calls. Symbols: *p < 0.05, **p < 0.01, a significant difference between vehicle- and poly(I:C)-exposed males (planned comparisons).

Moreover, there were no significant differences between poly(I:C)- and vehicle-exposed rats in the number of USV within call categories (treatment × sex × call type interaction: F[3,244] = 0.61, ns; Fig. 4a). However, a detailed analysis of acoustic parameters of individual call types revealed significant differences in the duration (p = 0.0273, Tukey HSD post hoc test following a significant treatment effect: F[1,61]  = 8.79, p = 0.0043; Fig. 4b) and bandwidth (p = 0.0345, Tukey HSD post hoc test following a significant treatment effect: F[1,61]  = 5.53, p = 0.0219; Fig. 4b) of high frequency modulated (HFM) calls. In addition, planned comparisons showed significant increases in the duration (t = 2.95, p = 0.0045; Fig. 4b) and bandwidth (t = 2.26, p = 0.0274; Fig. 4b) and a trend toward a raised peak frequency (t = 1.88, p = 0.0639; Fig. 4b) in males but not females.

**Figure 5:**
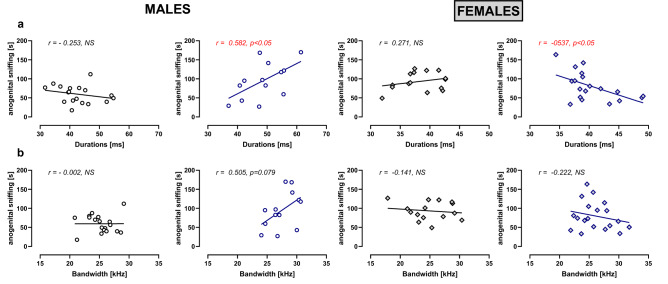
Correlation between anogenital sniffing and the acoustic parameters of high frequency modulated (HFM) USVs. A Pearson correlation coefficient (r) was calculated for the relationship between the time spent on anogenital sniffing and the duration **(a)** and bandwidth **(b)** of HFM calls.

Correlation analyses were performed to test whether increased anogenital sniffing behavior in poly(I:C) males may be related to the acoustic parameters of HFM calls. As illustrated in Fig. 5, there was a significant positive correlation for the duration (r = 0.582, p = 0.0371; Fig. 5a) and a tendency toward a positive correlation for the bandwidth (r = 0.505, p = 0.0786; Fig. 5b) of HFM calls in poly(I:C) males. There were no significant correlations in vehicle-treated males and females, whereas a negative correlation was observed for the duration parameter in poly(I:C) females (r = -0537, p = 0.0178; Fig. 5a).

### Tickling-induced USVs

**Figure 6:**
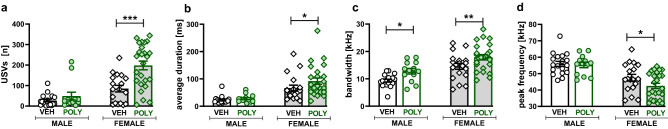
Tickling-induced vocalizations: total USV emission. Poly(I:C) exposure increased the total number (**a**), average duration **(b),** and bandwidth **(c)** but decreased the peak frequency (**d**) of emitted calls. Data are presented as a mean ± SEM. Symbols: *p < 0.05, **p < 0.01, ***p < 0.001, a significant difference between vehicle- and poly(I:C)-exposed rats within the given sex (planned comparisons).

Poly(I:C) exposure increased the total number of calls emitted during tickling in a sex-dependent manner (treatment × sex: F[1,70]  = 6.13, p = 0.0157; Fig. 6a). While poly(I:C) males did not differ from controls, enhanced vocalizations were demonstrated in females (t = 4.73, p < 0.0001, planned comparisons). Poly(I:C) also broadened the bandwidth of USVs (p = 0.0001, Tukey HSD post hoc test following a significant treatment effect: F[1,70]  = 14.16, p = 0.0004; Fig. 6c). These differences were noted in males and females (t = 2.35, p = 0.0213 and t = 3.04, p = 0.0033, respectively, planned comparisons). Moreover, planned comparisons revealed that poly(I:C) females’, but not males’, vocalizations were longer (t = 2.01, p = 0.0486; Fig. 6b) and emitted on lower frequencies (t = 2.15, p = 0.0347; Fig. 6d).

**Figure 7:**
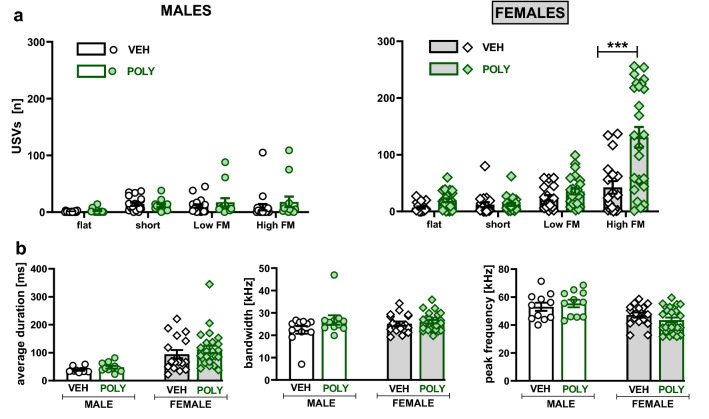
Tickling-induced vocalizations: USV categories. Poly(I:C) exposure increased the number of high frequency modulated (HFM) USVs in females (**a**) but did not change the acoustic characteristics of HFM sounds (**b**). Data are presented as a mean ± SEM of the number of a given call type (**a**) and average duration, bandwidth, and peak frequency (**b**) of the HFM calls. Symbols: ***p < 0.001, a significant difference between vehicle- and poly(I:C)-exposed females (planned comparisons).

There was also a sex-specific effect of poly(I:C) on the number of USV within call categories (treatment × sex × call type interaction: F[3,283] = 5.46, p = 0.0012; Fig. 7a). Accordingly, poly(I:C) exposure increased the number of HFM USVs only in females (t = 8.58, p < 0.0001, planned comparisons; Fig. 7a). However, poly(I:C) had no effects on the acoustic parameters of HFM calls (Fig. 7b).

### Olfactory preference test

**Figure 8:**
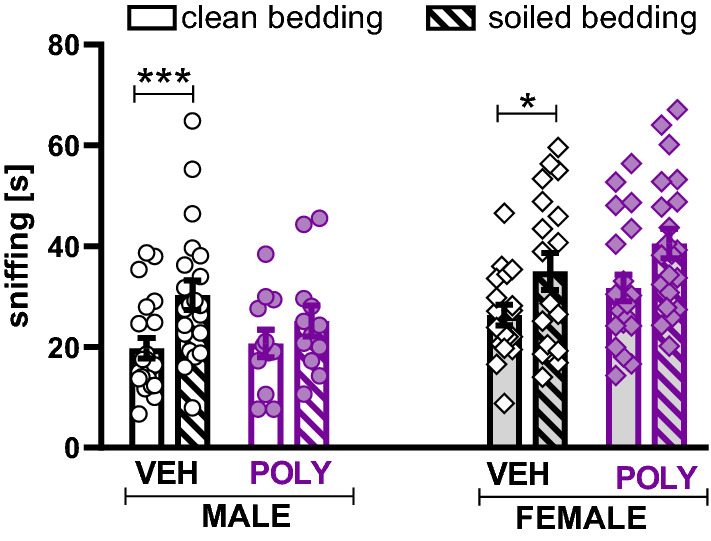
Olfactory preference test. Poly (I:C) exposure reduced preference for social stimuli. Data are presented as a mean ± SEM of time spent sniffing clean and soiled bedding. Symbols: *p < 0.05,***p < 0.001, a significant difference between clean and soiled bedding exploration in a given group (paired Student’s t-test).

As illustrated in Fig. 8, control animals preferred social odor, as revealed by the longer time spent sniffing soiled versus clean bedding (t = 4.01, p = 0.0007, and t = 2.33, p = 0.0324, paired Student’s t-test, for males and females, respectively). However, this preference was reduced in poly(I:C) exposed males (t = 1.17, p = 0.2681) and, to a lesser extent, in poly(I:C) females (t = 1.86, p = 0.0774).

### Locomotor activity and repetitive behaviors

**Figure 9:**
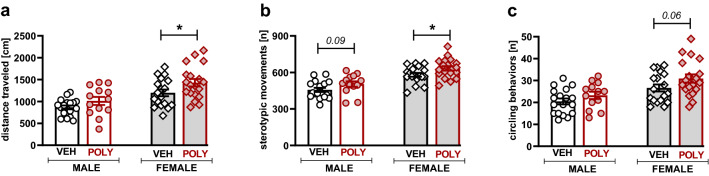
Locomotor activity. Poly(I:C) exposure increased the distance traveled (**a**), repetitive movements (**b**), and episodes of circling behavior (**c**). Data are presented as a mean ± SEM. Symbols: *p < 0.05, a significant difference between vehicle- and poly(I:C)-exposed females (planned comparisons).

Poly(I:C) exposure enhanced rats’ locomotor activity (p = 0.0038, Tukey HSD post hoc test following a significant treatment effect: F[1,64]  = 6.12, p = 0.016; Fig. 9a), increased the number of repetitive movements (p = 0.0012, Tukey HSD post hoc test following a significant treatment effect: F[1,64]  = 7.50, p = 0.0079; Fig. 9b) and increased the number of episodes of circling behavior (p = 0.0124, Tukey HSD post hoc test following a significant treatment effect: F[1,64]  = 4.49, p = 0.0378; Fig. 9c). Planned comparisons revealed significant differences between vehicle and poly(I:C) groups only in females (t = 2.26, p = 0.0272, and t = 2.15, p = 0.035, for the distance traveled and repetitive movements, respectively; Fig. 9a–c).

### ELISA

**Figure 10:**
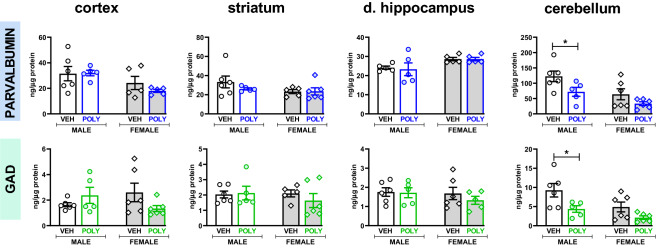
Protein levels of parvalbumin and GAD67. Poly(I:C) exposure decreased the expression of parvalbumin and GAD67 in the cerebellum. *p < 0.05, a significant difference between vehicle- and poly(I:C)-exposed males (planned comparisons).

As illustrated in Fig. 10, prenatal exposure to poly(I:C) reduced the protein levels of parvalbumin (p = 0.0103, Tukey HSD post hoc test following a significant treatment effect: F[1,19]  = 7.44, p = 0.0133) and GAD67 (p = 0.0046, Tukey HSD post hoc test following a significant treatment effect: F[1,19]  = 9.84, p = 0.0054) in the cerebellum. Analysis of treatment differences within the given sex revealed that significant decreases were observed only in males (t = 2.35, p = 0.0297 and t = 2.76, p = 0.0123, for parvalbumin and GAD67, respectively).

There were no significant treatment effects on parvalbumin and GAD67 protein expression in any other examined brain regions.

## Discussion

The present study demonstrated that prenatal poly(I:C) exposure altered the pattern of rats’ socio-communicative behaviors in a sex-specific manner. Accordingly, poly(I:C)-exposed males demonstrated increased anogenital sniffing behaviors. Poly(I:C) also affected the acoustic characteristics of USVs emitted by males during social interactions. Interestingly, the durations of high frequency modulated calls were positively correlated with the time of anogenital sniffing behavior. Poly(I:C) males also demonstrated impaired social odor discrimination deficits. While poly(I:C) females did not differ from controls in socio-positive behaviors, they displayed aggression during the social encounter. They were also more reactive to somatosensory stimulation, as revealed by increased USV emission during tickling. Furthermore, the locomotor pattern of poly(I:C) animals was characterized by repetitive movements, and these abnormalities were more pronounced in females. Finally, poly(I:C) reduced parvalbumin and GAD67 expression in the cerebellum, with significant changes noted in males.

Detailed analysis of the distinct types of social behavior showed increased anogenital sniffing in poly(I:C)-treated males. Rodents typically sniff the anogenital regions of conspecifics to decode identity^9^. In this sense, longer time spent on anogenital sniffing may suggest difficulties accepting a novel rat as familiar and consequently impaired social recognition. In line with this assumption, oxytocin and vasopressin reduced anogenital sniffing, implying increased familiarity in the presence of strangers, and this effect corresponded with enhanced social recognition^24^. Moreover, it has been proposed that decreases in sniffing serve as appeasement signals during rats’ social interactions, and the failure to decrease their sniffing frequency facilitates agonistic behavior by dominant rats^25^. Therefore, it cannot be excluded that the demonstrated in a current study behavioral pattern may suggest a reduced ability to form a social hierarchy in poly(I:C) males. This impairment could purportedly result from the previously demonstrated deficits in juvenile social play that hampered establishing proper adult hierarchical relations. However, while sniffing behavior is considered a useful tool for studying hierarchical interactions^26^, the complex nature of dominant behaviors requires cross-validation with other hierarchy assessment tests to accurately determine social rank behavior.

Considering previously demonstrated social deficits in adolescent rats^15^, it may be surprising that poly(I:C)-evoked changes in adult rats’ behavior appeared quite subtle. However, several previously published studies using this MIA model demonstrated a lack of evident social deficits during adulthood. For example, Gray et al.^27^ reported no poly(I:C) effects on social exploration and other behavioral measures in the social interaction test in both males and females. Similarly, males’ prosocial behaviors (including body and anogenital sniffing, crawling, or following) were not modified by poly(I:C) in the studies by Goh et al.^28^ and Chamera et al.^29^. On the contrary, Osborne et al.^30^ demonstrated reductions in total interaction time in poly(I:C) adult offspring and this deficit was evident regardless of sex^31^.

While most studies focused on prosocial, non-aggressive behaviors, increased aggression was also demonstrated in poly(I:C) males^29^. Surprisingly, we observed MIA-induced aggression in females but not males. While the source of these discrepancies is unknown, several mechanisms may exert sex-specific control over aggression^32^. For example, vasopressin, which may contribute to the sexually dimorphic effects of MIA^33^, is also involved in the sex-specific modulation of aggression in rodents. So, it can be concluded that deficits in social domains may be differentially expressed in males and females and may not simply be manifested as reductions in behavior.

It is worth mentioning that most of the published reports on poly(I:C)-induced social deficits have utilized a three-chamber test. This paradigm is based on an easily quantifiable sociability measure reflected as a tendency to spend more time in the compartment with an unfamiliar conspecific than in the empty compartment^34^. As this test does not require direct social interaction, the measured parameters may reflect social preference or interest and not necessarily the ability to be engaged in a social encounter. Therefore caution is needed when comparing these two paradigms. Although we did not observe an apparent reduction in social behaviors during the interaction test, poly(I:C) reduced sociability in late adolescent or adult mice^35–38^ and adult rats^39,40^ (but see also^41–43^ for the opposite results). While most studies focused only on males, there are still some reports on sociability deficits in females^38,44^.

Nevertheless, these findings from a three-chamber test confirm our results demonstrating impaired social odor preference in poly(I:C) rats. The weaker interest in social stimuli appeared not to be due to impaired olfaction because MIA offspring did not display an olfactory deficit, as measured in the olfactory sensitivity test^35^. Alternatively, reduced novelty-seeking behavior may result from enhanced anxiety, as demonstrated in the poly(I:C) model^36^ (but see^29^ for the opposite results).

Literature data suggest that poly(I:C) exposure may affect social recognition memory. Accordingly, in the study by Talukdar et al.^39^, poly(I:C) males exhibited impaired social novelty preference expressed as a reduced natural tendency to spend more time with a novel rat than a familiar one. Unfortunately, we did not incorporate the stage directly assessing social recognition in our odor preference test. However, this assumption fits well with enhanced anogenital sniffing as a signature of poor recognition of conspecifics’ identity.

Another hallmark of autistic-like deficits may be poor ultrasonic communication during social encounters^8^. Accordingly, our previous study demonstrated deficits in social play behaviors in poly(I:C) adolescent males accompanied by reduced USV emission^15^. However, these communication deficits did not persist until adulthood, as our current study did not demonstrate any poly(I:C)-evoked quantitative changes in the call number in adult animals. In line with our data, the study by Scott et al.^45^ also did not reveal significant changes in the total number of calls or the proportion of call type categories in poly(I:C) male rats. A similar conclusion emerges from another MIA model based on administering the bacterial endotoxin lipopolysaccharide (LPS). While USV emission decreased in LPS-exposed adolescent male offspring^46,47^, the differences were no longer observed in adulthood^47^.

Furthermore, analysis of acoustic call features revealed that poly(I:C) exposure increased USV peak frequencies. Our previous studies in the poly(I:C) model demonstrated that calls of an elevated frequency were emitted by pups when separated from their mothers^48^ and by adolescent rats during social play^15^. Although the physiological meaning of this acoustic feature is unknown, it may be interpreted as a sign of life-long communication abnormalities, at least in this model.

We did not demonstrate any other poly(I:C)-evoked changes when analyzing the parameter of calls as total; the differences, however, emerged when assessing the acoustic characteristic of distinct call types. Accordingly, significant increases in the duration and bandwidth of high frequency modulated calls were observed in poly(I:C) males. The result of Scott et al.^45^ confirms our observation demonstrating that calls from poly(I:C) animals were longer in duration than control calls. These changes in call duration were specific to highly modulated USV categories, including complex and trill subtypes^45^. The latter authors concluded that the extended call duration might affect temporary call distributions, revealed as a tendency for poly(I:C) animals to generate pairs of calls with short inter-call intervals. Interestingly, the sequential structure of USV communications of MIA animals was also changed in that study^45^. Whether these aspects of communication are also affected in our experimental conditions awaits further studies.

Since the observed in our study changes manifested in increased anogenital sniffing behavior and altered acoustic features of HFM calls, the correlation analyses between these parameters were performed. The anogenital sniffing in poly (I:C) males was positively correlated with the duration of HFM calls, whereas a negative correlation was observed for these parameters in poly(I:C) females. Even though we lack a clear interpretation of this finding, the observed correlations may support the different nature of socio-communicative deficits in males and females.

Rats also emit 50-kHz calls when they are stimulated (“tickled”) in a playful way by the human. The tickling-induced USVs reflect positive affect similar to this commonly accompanying social play^13^. Although this phenomenon has been initially described in juvenile rats, manual stimulation also evokes vocalizations in adult rats^16^. In addition, tickling-induced USVs are sensitive to drug treatment^49^ and stress^16^ and are altered in a neurodevelopmental model of schizophrenia^50^. Our results demonstrate that MIA can also affect USV emission during tickling. Interestingly, poly (I:C) exposure increased the call number in a sex-dependent manner, as enhanced vocalizations were demonstrated only in females. Whether this effect reflects enhanced positive affect or rewarding properties of manual stimulation seems disputable. Firstly, the animal’s response to tickling develops gradually and stabilizes within a week of stimulation^16^. Our study used a 2-day procedure to avoid a confounding factor that might influence autistic-like phenotype per se. Consequently, there was a relatively low basal level of USV production, suggesting that rats had not yet developed a positive reaction to tickling. In this light, females’ increases in USVs may also reflect an aberrant somatosensory reaction to touch. Sensory abnormalities, including aberrant tactile sensitivity, are a common feature of ASD^51^. For example, some ASD patients display exaggerated responses to touch^52^. Sensory over-responsiveness has also been demonstrated in animal ASD models^51^, including MIA models^53^. Since tactile abnormalities may contribute to the core ASD symptoms, for example, social dysfunctions, it can not be excluded that elevated aggression in poly(I:C) females might be linked to their tactile sensory phenotypes.

Repetitive and stereotyped behavior patterns, which comprise the second core symptom of ASD, are also reported in MIA models. In the current study, we also observed repetitive movements that characterized the locomotor pattern of poly(I:C) animals. Unfortunately, we cannot precisely define what kind of repetitive behavior was scored by the Opto-Varimex-4 system, as the only measure was the number of repeated breaks of the same beam. Thus, one may assume that repetitive-like movements assessed may not necessarily result from goal-directed behavior but, for example, from hyperlocomotion or enhanced circling behavior that also characterized poly(I:C) animals.

In our previous study, poly(I:C)-exposed adolescent rats exhibited repetitive digging behaviors, and repetitive movements characterized their locomotor pattern^15^. Interestingly, females manifested these abnormalities to a greater extent than males. In line with the poly(I:C) model, enhanced locomotion was demonstrated in LPS-exposed adolescent females but not males^47^. In the latter study, however, this deficit was no longer evident in adulthood. On the contrary, in the current study, adult poly(I:C) females’ behavior was still characterized by hyperlocomotion and repetitive behaviors. These results further support the sex-specific manifestation of autistic symptoms and the necessity of including females in ASD studies.

GABAergic alterations have been consistently connected to ASD. For example, brain tissue samples from ASD patients show reduced expression of the rate-limiting enzymes in GABA synthesis, glutamic acid decarboxylase (GAD65), and 65-kDa (GAD67) proteins^54^. According to clinical data, the protein expressions of GAD65 and GAD67 were reduced in animal models of ASD^55^, and GAD67-deficient mice displayed behavioral abnormalities reminiscent of ASD-like pathologies^56^. Reduced GAD67 levels were also reported in the medial prefrontal cortex of poly(I:C)–exposed adult offspring^57^. A mechanism whereby MIA can induce such GABAergic impairments in the offspring may be related to epigenetic modifications of the promotor region of GAD1 (which encodes GAD67) and GAD2 (which encodes GAD65)^37^. There is also evidence that one of the features of ASD is decreased expression of parvalbumin, a calcium-binding protein present in a subpopulation of GABAergic interneurons^58,59^. These findings are also supported in animal studies; for example, parvalbumin knockout mice displayed behavioral phenotypes relevant to the core ASD symptoms^60^. Reduced parvalbumin expression was also reported in ASD models, including the poly(I:C) model^61,62^. In line with the literature data, our study also demonstrated reduced levels of GAD67 and parvalbumin; however, these changes were observed only in the cerebellum.

Existing data suggest that the cerebellum may play a critical role in the pathogenesis of ASD^63,64^. Structural, functional, and neurochemical cerebellar abnormalities have been demonstrated in clinical and preclinical ASD studies^65^, including the poly(I:C) model^66^. For example, ASD patients exhibited decreased expression of parvalbumin and GAD67 in cerebellar Purkinje cells^59,67^. Thus, it is not surprising that cerebellar reductions of these GABAergic markers have also been observed in the current study. Interestingly, significant reductions were demonstrated only in males. This finding may corroborate the clinical observations that cerebellar neuropathology in autism is not only region-specific but also sex-specific^68^. In line with clinical data, prenatal valproic acid exposure led to specific Purkinje cell loss within the cerebellum^69,70^. While the extent of loss tended to be larger in males, there were also sex-dependent regional differences. Likewise, a significant reduction of the number of Purkinje cells in Crus I was demonstrated only in males, and this cell loss correlated with social interaction deficits^70^. In line with this observation, it has been proposed that cerebellar dysfunction may affect cerebello-cortical circuitry leading to the core ASD symptoms^71^.

Sex-dependent effect of MIA on parvalbumin expression has also been demonstrated in the cortical and hippocampal brain regions^72,73^; however, other authors showed a reduced number of cortical parvalbumin-positive interneurons in both sexes^47^. Nevertheless, we did not detect any changes beyond the cerebellum. It cannot be excluded that differences would be found in the number of parvalbumin-positive GABAergic interneurons or parvalbumin immunoreactivity. Similarly, we found GAD67 changed only in the cerebellum in contrast to several works showing altered GAD67 expression in other brain regions. For example, GAD67 mRNA expression was decreased in the prelimbic region of the prefrontal cortex^74^, and GAD67 protein expression was reduced in the dorsal hippocampus^75^ in adult poly(I:C) offspring. Likewise, examining mRNA expression or using different protein analysis techniques (e.g., Western blotting) could presumably help reveal changes in the GAD67 level in the current study.

In summary, poly(I:C)-evoked deficits are also demonstrated in adult rats; however, in contrast to adolescent rats, these changes are not simply manifested as reductions in behavior. Poly(I:C) treated rats displayed sex-specific differences across a domain of social behaviors, and several changes in their USVs were correlated with these behaviors. This sex-dependent pattern of changes further supports the necessity of including females in ASD studies.

## Materials and methods

### Animals

Pregnant dams (Sprague–Dawley rats, N = 12) were obtained from Charles River (Sulzfeld, Germany) on gestation day (G.D.) 9–10. They were housed individually in polycarbonate cages: 26.5 (width) × 18 (height) × 42 (length) cm. On postnatal day (PND) 21, pups were weaned and separated by sex and litter into groups of 3–5 rats. Poly(I:C) and saline-exposed offspring were housed in separate cages. Females and males were housed in different temperature-controlled (21 ± 1 °C) and humidity-controlled (40–50%) colony rooms under a 12/12 h light/dark cycle (lights on at 06:00 h). Food and water were available ad libitum. Behavioral testing was performed during the light phase of the light/dark cycle. The experiments were conducted in accordance with the European Guidelines for animal welfare (2010/63/E.U.) and were approved by the II Local Ethics Committee for Animal Experiments at the Maj Institute of Pharmacology, Polish Academy of Science, Krakow, Poland (permission number: 203/2017). This study was performed in accordance with ARRIVE guidelines.

### Poly(I:C) administration and experimental schedule

On GD 15, the dams were injected intraperitoneally (i.p.) with either physiological saline (vehicle) (N = 6) or poly(I:C) at a dose of 5 mg/kg (N = 6). Poly(I:C) (P9582, Sigma-Aldrich, Poznan, Poland) was dissolved in physiological saline. Both poly(I:C) and vehicle were administered at a volume of 2 ml/kg. The dose and time of poly(I:C) administration were based on previous reports demonstrating autistic-like behaviors, including social and communicative abnormalities^15,48^. This schedule of poly(I:C) administration was effective in inducing immune activation, as revealed by elevated maternal serum cytokine concentrations^40^.

There were no effects of treatment on gestation length and litter size (N = 13 and N = 12, for control and poly(I:C) litters, respectively), but there was a prevalence of females in poly(I:C) offspring (male/female ratio for six litters: ≈ 0.63 for poly(I:C) animals and ≈ 1.08 for controls). As previously demonstrated^15^, the rats prenatally exposed to poly(I:C) appeared healthy and could not be behaviorally distinguished from the controls. They also did not differ from control animals in body weight (Fig. S1; Supplement 1).

In total, 41 males and 38 females were born from 6 vehicle-treated dams, and 26 males and 41 females from 6 poly(I:C)-treated dams. On PND ≈ 80, all animals were subjected to social interaction test. One week later, half of the rats were used for the olfactory preference test, and locomotor activity was measured in another half. At the end of behavioral testing, the tickling procedure was conducted on the randomly chosen half of the animals. A schematic of the experimental design is shown in Fig. S2 (Supplement 2). To minimize the risk of a litter effect, offspring were randomly and equally distributed across these procedures. The method sections provide a detailed description of the number of animals per group in each experiment.

### Social interaction test

The test was performed as previously described^76,77^. Social behavior was observed in same-sex, same-treatment pairs of rats placed in a dimly illuminated (15 Lux) open field (dimensions: 57 × 67 × 30 cm, material: black Plexiglas). One day before the test, the rats were individually placed in the open field for 5 min to adapt them to the testing area. Then, the animals were weighed, and the backsides of one-half of the animals were marked with a Pentel permanent marker. On the test day, two unfamiliar rats of matched body weight (± 5 g) were placed in the open-field arena, and their behaviors were recorded for 10 min using a Sony light-amplification CCD camera placed above the arena and connected to a P.C. running a Noldus MPEG recorder 2.1. An experimenter blind to the treatment conditions analyzed the videos offline using Noldus Observer® XT, version 10.5.

The measured social behaviors included: sniffing (the rat sniffs the body of the conspecific), anogenital sniffing (the rat sniffs the anogenital region of the conspecific), social grooming (the rat licks and chews the fur of the conspecific), climbing (the rat climbs over the back of the conspecific/stands on the back of the conspecific) and following (the rat moves toward and follows the conspecific). Moreover, aggressive behaviors scored included: biting, boxing, chasing, aggressive grooming, clinching, lateral threatening, and keeping down. The duration and number of episodes of social behavior were measured for each rat separately and summed to give a total score for each pair of animals.

The number of pairs used in the analysis was: N = 18 (vehicle males), N = 13 (poly(I:C) males ), N = 15 (vehicle females), and N = 19 (poly(I:C) females). Seven pairs (vehicle males: two, vehicle females: four, and poly females: one) were excluded from the analysis due to failure in the Noldus or Avisoft recording.

### Tickling-induced USVs

The manual somatosensory stimulation (tickling) was conducted as previously described^16,50^ and consisted of gentle holding of the rat on its back with the investigator’s left hand and rapid right-hand finger movements across the ventral body surface of the animal for 15 s, followed by 5 s of no stimulation. The stimulation cycle was conducted for a total of 3 min. Each rat experienced one day of tickling for habituation to the tickle procedure. An identical procedure was used on the second day, except an ultrasound microphone was ON and recorded USVs.

The number of rats used in the analysis was: N = 18 (vehicle males), N = 13 (poly(I:C) males), N = 18 (vehicle females), and N = 25 (poly(I:C) females).

### USV recording

As previously described^15,76,77^, the rats’ vocalizations were recorded during the entire test session (i.e., 10 min or 3 min) using a frequency response range of 2–200 kHz microphone (UltraSoundGate Condensor Microphone CM16/CMPA, Avisoft Bioacoustics, Berlin, Germany) suspended 25 cm above the floor of the test area. Microphone signals were fed into an UltraSoundGate 416H (Avisoft Bioacoustics, Berlin, Germany) before the analog signal was digitized with a sampling rate of 200 kHz and a 16-bit resolution. Acoustic data were recorded using Raven Pro: Interactive Sound Analysis Software, version 1.5 (The Cornell Lab of Ornithology Bioacoustics Research Program, Ithaca, NY, USA). The calls were manually marked on the computer screen and counted by an experienced user, blind to the treatment, using the Raven Pro software. The spectrograms were generated with a fast Fourier transform (FFT)-length of 512 points and a time-window overlap of 75% (100% frame, Hamming window).

The 50-kHz USVs were further manually divided (based on their acoustic call features) into the following general types: short calls, flat calls with a near-constant frequency, and frequency-modulated calls. The frequency-modulated calls were subsequently classified as low frequency modulated calls (complex calls, ramp, and inverted-U calls) and high frequency modulated calls (mostly trills, but also multi-step, step-up, step-down, and composite calls). We also analyzed the following USV features: a) the call duration (length of the call, measured in milliseconds), b) the bandwidth (the difference between the highest and lowest frequencies, a measure of frequency modulation, expressed in kHz), and c) the peak frequency (the frequency in kHz at which maximal energy occurs within the selection). The 22-kHz alarms were excluded from the further analysis due to their negligible distribution (≥ 1.6%). The number of analyzed samples was the same as for the social interaction test and manual tickling procedure.

### Olfactory preference test

The procedure was conducted according to the previously published protocol^77^. Two bowls (an internal diameter of 8 cm and a depth of 4 cm) were placed on one side of the open-field apparatus (the same one used for the social interaction test). Each bowl contained one of the following odor stimuli: (a) clean bedding (clean sawdust) and (b) same-sex soiled bedding (sawdust collected from the cages of females or males, respectively). The bedding samples were obtained from cages of unfamiliar, group-housed, sexually inexperienced males or females on the fifth day following the previous bedding change. This five-day period was necessary to soak the wood shavings with odors of feces, urine, and pheromones.

One day before the test, rats were habituated to the empty apparatus for 5 min. On the test day, the rats were exposed to clean and soiled bedding. Bowl location (right or left positioning) was counterbalanced between rats. The test started with the subject in the center of the apparatus. In a 5-min test, we recorded the time the rat actively sniffed each bowl. Sniffing was defined as the subject’s nose directly contacting the bedding or the bowl. After each measurement, the floor and bowls were cleaned and dried.

The behavior of the rats was recorded using a Tayama camera (C3804-01A1, Katowice, Poland) placed above the open field and connected to the Any-maze® tracking system (Stoelting Co., USA, Illinois). An experimenter blinded to the treatment conditions manually assessed the exploration time. The data from any rat spending less than 5 s exploring the bedding samples were removed from the analyses. These exclusion criteria were set to limit confounding factors related, for example, to general changes in exploratory activity.

The number of animals in a given group was: N = 21 (vehicle males), N = 12 (poly(I:C) males), N = 18 (vehicle females), N = 21 (poly(I:C) females). The analysis excluded two rats (one poly male and one vehicle female) due to low exploration time.

### Locomotor and repetitive activity

Spontaneous locomotor activity was measured automatically in Opto-Varimex-4 Auto-Tracks (Columbus Instruments, OH, USA) located in the sound-attenuated and ventilated boxes. The Auto-Track System sensed the motion with a grid of infrared photocells (16 beams per x- and y-axis) surrounding the arena. The data collected every 1 min during a 10-min session are presented as (a) the total distance traveled, (b) the number of repetitive movements (defined as the number of repeated breaks of the same beam), and (c) the total number of episodes of circling behavior (the total number of clockwise and counter-clockwise rotations).

The number of animals in a given group was: N = 18 (vehicle males), N = 13 (poly(I:C) males ), N = 18 (vehicle females), N = 19 (poly(I:C) females). Due to technical reasons, four rats were excluded from the analysis (two vehicle males, one vehicle female, and one poly(I:C) female).

### Enzyme-linked immunosorbent assays (ELISA)

One day after behavioral testing, poly(I:C)- and vehicle-exposed rats of each sex were sacrificed via decapitation, and the brains were removed. Samples of the prefrontal cortex, striatum, dorsal part of the hippocampus, and cerebellum were quickly frozen on dry ice and stored at − 80 °C. Then, the tissues were moved to −20 °C and homogenized on ice (TissueLyser, Qiagen, USA) in RIPA buffer with cOmplete™ Mini Protease Inhibitor Cocktail (11836153001, Roche). The homogenates were centrifuged for 30 min at 15.500×*g* at 4 °C. Supernatants were collected for future analysis.

Bradford reagent (B6916, Sigma Aldrich) was used to determine the total protein concentration of each sample. Levels of glutamic acid decarboxylase 1 (GAD1) and Parvalbumin Alpha protein were assessed using ELISA kits (Cat. E1353Ra for GAD1 and E2521Ra for parvalbumin, Bioassay Technology Laboratory), according to the manufacturer protocol. After the reaction was terminated, the absorbance was measured at 450 nm using the SynergyMx apparatus (BioTek, Vermont, USA). The analyses of the ELISA results were performed on raw data expressed as the mean arbitrary absorbance units per well as ng of the GAD1 and parvalbumin per 1 μg of total protein.

The number of animals in each given group was: N = 5–6. The sample size was calculated based on the previously published study^77^ using G*Power software, version 3.1.9.4 (Franz Faul, Universität Kiel, Kiel, Germany). The calculated sample size was based on an alpha error of 0.05 and 85–90% power.

### Statistics

Unless stated otherwise, we used a priori planned comparisons of Least Squares means to compare vehicle and poly(I:C) conditions within a given sex. Moreover, the overall treatment effects were tested by ANOVA analysis. When there was a significant main effect of treatment, we used the Tukey HSD post hoc tests to assess overall differences between vehicle- and poly(I:C)-exposed groups.

The arcsine-transformed percentage of time and the number of episodes of behavior were subjected to three-way ANOVAs with treatment (vehicle vs. poly(I:C)), sex (male vs. female), and type of behavior as between-subject factors. For the number and time of aggressive behaviors, Mann–Whitney U-test was used. The number of USVs was analyzed by a three-way ANOVA with treatment, sex, and call category as between-subject factors. Acoustic call features (duration, bandwidth, and peak frequency) were analyzed by two-way ANOVAs with treatment and sex as between-subject factors. Pearson’s product-moment method was used to correlate behavioral and USV measures. The differences in time spent sniffing clean vs. soiled bedding were analyzed by paired Student’s t-tests. Locomotor activity and ELISA data were analyzed by two-way ANOVAs with treatment and sex as between-subject factors.

The effect size was estimated using partial eta squared (ŋp2). The normality of data distribution was evaluated by the Kolmogorov–Smirnov test. Statistical significance was set at p < 0.05. The statistical analyses were performed using Statistica 12.0 for Windows. Detailed ANOVA results and the effect sizes are presented in Table S3 (Supplement 3).

### Identification of the estrous cycle phase

After behavioral testing, vaginal cytology samples were collected, and the estrous cycle stage was determined by examining the appearance and abundance of cells in vaginal samples, as previously described in detail by Potasiewicz et al.^77^. In line with our previous study^48,77^, we did not observe the influence of the estrous cycle on any measured parameters (data not shown).

## Supplementary Information

Supplementary Information.

## Data Availability

All data generated during this study are included in this article and its supplementary file.
