# Supplementary material for: Maternal immune activation affects socio-communicative behavior in adult rats
Source: Sci Rep. 2023 Feb 2;13:1918. doi: 10.1038/s41598-023-28919-z (PMC9894913; doi:10.1038/s41598-023-28919-z)
Supplement: Supplementary file 1 — Supplementary Information. [file 41598_2023_28919_MOESM1_ESM.docx]

**Supplements:**

**Supplement 1: Rats' body weight.**

**Supplement 2: A schematic of the experimental design.**

**Supplement 3: ANOVA results.**

**Maternal immune activation affects socio-communicative behavior in adult rats.**

Kinga Gzieło, Diana Piotrowska, Ewa Litwa, Piotr Popik, Agnieszka Nikiforuk*

Maj Institute of Pharmacology Polish Academy of Sciences, Department of Behavioral Neuroscience and Drug Development, Kraków, Poland

**Supplement 1. Rats' body weight (PND=80).**

There were no differences between either vehicle- and poly(I:C)-treated rats or males and females.

**Figure S1. Body weight.**

Poly(I:C) exposure did not affect body weight in either males or females (treatment effect: F[1,126]=0.44, ns; treatment x sex interaction: F[1,126]=0.06, ns). Irrespectively of treatment, female rats weighed less than males (p<0.0001, Tukey HSD post hoc test following a significant sex effect: F[1,126]=1050.06, p<0.0001).

The number of animals used in the analysis was: N=36 (vehicle males), N=30 (vehicle females), N=26 (poly(I:C) males), and N=38 (poly(I:C) females).

**Supplement 2: A schematic of the experimental design.**

**
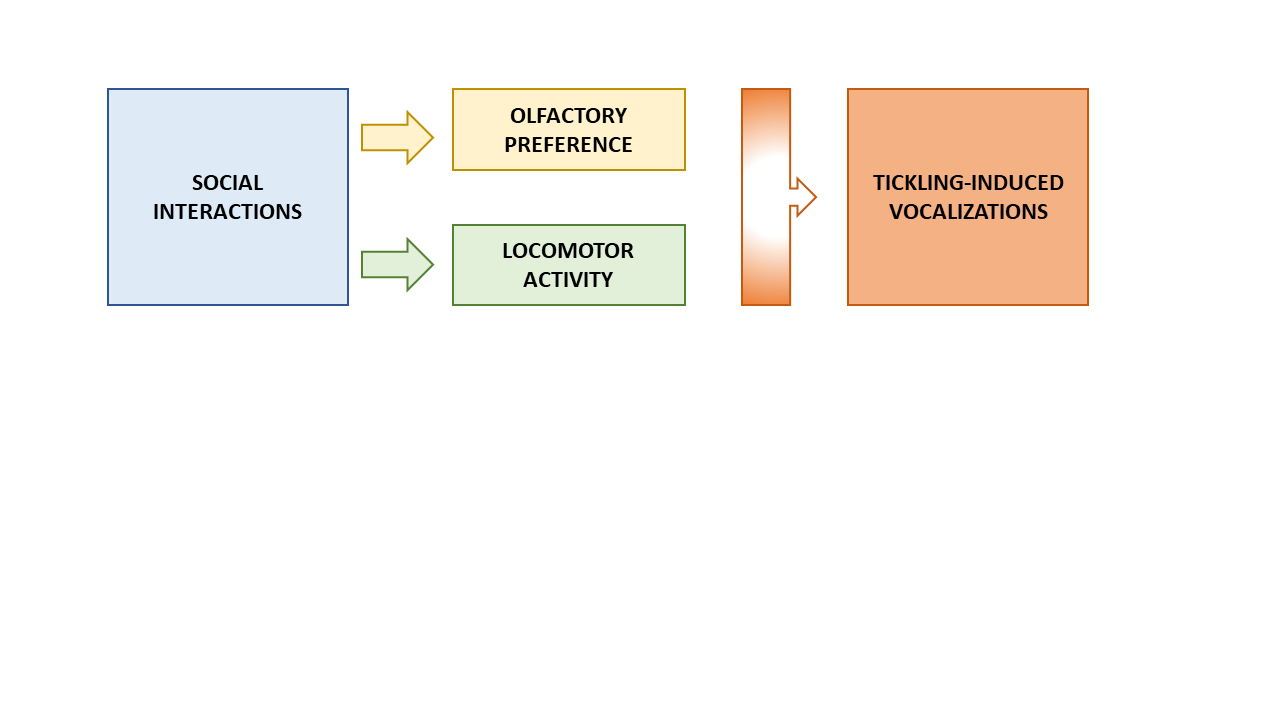
**

**Figure S2. A schematic of the experimental design.**

On PND ≈ 80, all animals were subjected to social interaction test. One week later, half of the rats were used for the olfactory preference test, and locomotor activity was measured in another half. After a subsequent week, the tickling procedure was conducted on the randomly chosen half of the animals.

**Supplement 3. ANOVA results**

**Table S3.1. Behavior during the social interaction test**

1. **Time (expressed as a percentage)**

1. **The number of episodes**

**Table S3.2. Ultrasonic vocalizations during the social interaction test.**

1. **Total USV emission**

**i). USV number**

**ii). Time**

**iii). Band**

**iv). Peak frequency**

1. **The number of call types**
2. **The acoustic characteristics of high frequency modulated (HFM) calls.**

**i). Time**

**ii). Band**

**iii). Peak frequency**

**Table S3.3. Ultrasonic vocalizations during tickling.**

1. **Total USV emission**

**i). USV number**

**ii). Time**

**iii). Band**

**iv). Peak frequency**

1. **The number of call types**

1. **The acoustic characteristics of high frequency modulated (HFM) calls.**

**i). Time**

**ii). Band**

**iii). Peak frequency**

**Table S3.4. Locomotor activity.**

1. **The distance traveled**

1. **Stereotypic-like movements**

1. **episodes of circling behavior**

**Table S3.5. ELISA.**
